# Artificial Intelligence Assisted Topographic Mapping System for Endoscopic Submucosal Dissection Specimens

**DOI:** 10.3389/fmed.2022.822731

**Published:** 2022-06-09

**Authors:** Yu Xiao, Zhigang Song, Shuangmei Zou, Yan You, Jie Cui, Shuhao Wang, Calvin Ku, Xi Wu, Xiaowei Xue, Wenqi Han, Weixun Zhou

**Affiliations:** ^1^Department of Pathology, Peking Union Medical College Hospital, Chinese Academy of Medical Sciences and Peking Union Medical College, Beijing, China; ^2^Department of Pathology, The Chinese PLA General Hospital, Beijing, China; ^3^Department of Pathology, National Cancer Center/National Clinical Research Center for Cancer/Cancer Hospital, Chinese Academy of Medical Sciences and Peking Union Medical College, Beijing, China; ^4^Institute for Interdisciplinary Information Sciences, Tsinghua University, Beijing, China; ^5^Thorough Images, Beijing, China; ^6^Department of Gastroenterology, Peking Union Medical College Hospital, Chinese Academy of Medical Sciences and Peking Union Medical College, Beijing, China

**Keywords:** endoscopic submucosal dissection, artificial intelligence, topographic mapping, diagnosis, pathology

## Abstract

**Background:**

Endoscopic submucosal dissection (ESD), a minimally invasive surgery used to treat early gastrointestinal malignancies, has been widely embraced around the world. The gross reconstruction of ESD specimens can facilitate a more precise pathological diagnosis and allow endoscopists to explore lesions thoroughly. The traditional method of mapping is time-consuming and inaccurate. We aim to design a topographic mapping system *via* artificial intelligence to perform the job automatically.

**Methods:**

The topographic mapping system was built using computer vision techniques. We enrolled 23 ESD cases at the Peking Union Medical College Hospital from September to November 2019. The reconstruction maps were created for each case using both the traditional approach and the system.

**Results:**

Using the system, the time saved per case ranges from 34 to 3,336 s. Two approaches revealed no significant variations in the shape, size, or tumor area.

**Conclusion:**

We developed an AI-assisted system that would help pathologists complete the ESD topographic mapping process rapidly and accurately.

## Background

Endoscopic submucosal dissection (ESD) is a well-accepted endoscopic resection method for removing early malignant gastrointestinal (GI) lesions. ESD can remove large and irregular superficial lesions en bloc while keeping the organs complete. It causes little damage to the patients and significantly improves their postoperative quality of life. Although the technique requires a steep learning curve, it is now widely used in Japan, China, and many nearby Asian countries, and is increasingly favored in Europe and the United States (1–3).

After the operation, the ESD specimens should be carefully evaluated by pathologists. Except for the diagnosis of the lesion, parameters such as the size, boundary, depth of infiltration, and lymphatic vascular invasion of the lesion should be evaluated accurately one by one. If there are high-risk factors for metastasis, further surgical treatment is required. The topographic mapping of the specimen is an essential step of pathological evaluation. It shows the size and shape of the lesion clearly and helps to judge the involvement of the cutting edge. Besides, reconstruction of the lesion and correlation of endoscopic changes could help endoscopists perform better treatments (4–6).

The traditional method of mapping is a tedious and time-consuming process. To map the specimen accurately, one has to mark the tumor area in the slides, then map each point of the entire area to the cutting lines on the gross picture proportionally. In particular, when the lesions are large and irregular, it may take a pathologist many hours to reconstruct one case (4, 7). With the rapid development of science and technology, artificial intelligence (AI) has gradually penetrated the medical field, especially in the fields of endoscopy, imaging, and pathology. In endoscopic diagnosis, studies successfully applied AI to the screening and monitoring of early cancer (8), detecting lesions easily overlooked by endoscopy (9) and diagnosing inflammatory bowel diseases (10). All previous study aimed to improve diagnostic efficiency and accuracy. In the field of pathology, AI has been used to detect tumor tissue, measure clinical outcomes, and predict molecular and genetic alterations (11). Our team also applied AI to identify GI tumors and achieved clinically applicable results (12). In this report, we established an AI-assisted automatic topographic mapping system for ESD specimens.

## Materials and Methods

### Case and Specimen Processing

We enrolled 23 continuous ESD specimens at Peking Union Medical College Hospital from September 2019 to November 2019. All cases were early tumors confirmed by biopsies and treated with en bloc ESD resection. There were nine cases of well-differentiated adenocarcinoma, four cases of moderately-differentiated adenocarcinoma, six cases of high-grade dysplasia, one case of low-grade dysplasia, one case of squamous cell carcinoma (esophageal), and two cases of well-differentiated neuroendocrine tumor.

After the ESD operation, all specimens are carefully extended by the endoscopist and pinned to the flexible plastic plate with fine steel needles. The specimen was then fixed in a 10% neutral buffer formalin solution for 12–48 h. Two photographs were taken before cutting: one is the original photograph with steel needles, and the other is a complete specimen photograph with the needles removed. The specimen was cut into tissue strips every 2 mm according to the standard procedure (1, 4). After being properly segmented according to size, they were grouped into embedding boxes to be paraffin-embedded, sectioned, and hematoxylin-eosin (HE) stained. The third photograph was taken after cutting the tissue into strips, and the fourth photograph was recorded after grouping. Each block is given a number, and the position of the tissue within the block is marked so that the same tissue strips can be used for mapping (7).

### Traditional Method of Mapping

All cases were mapped using traditional methods and the topographic mapping system, respectively. In the traditional way, the lesion area was marked on the slides with a marker pen as the pathologist read the slides. Then, attributes of the lesion were measured and recorded, including the total length of each strip, the length of the lesion area, and its position on the strip. The lesion area, of each tissue was then drawn on the photograph of the tissue strips according to the marked slides (i.e., the third photograph) in proportion to complete the reconstruction (7).

### Mapping With the System

All cases were also mapped using the system. First, the pathologist imported the gross photograph and the corresponding digital slides of tissue into the system. Then the pathologist marked the sampling sequence and the lesion area on the digital slides. After the two steps were completed, the system then automatically reconstructed the gross picture of the lesion.

### Overview of the Mapping System

**Figure 1:**
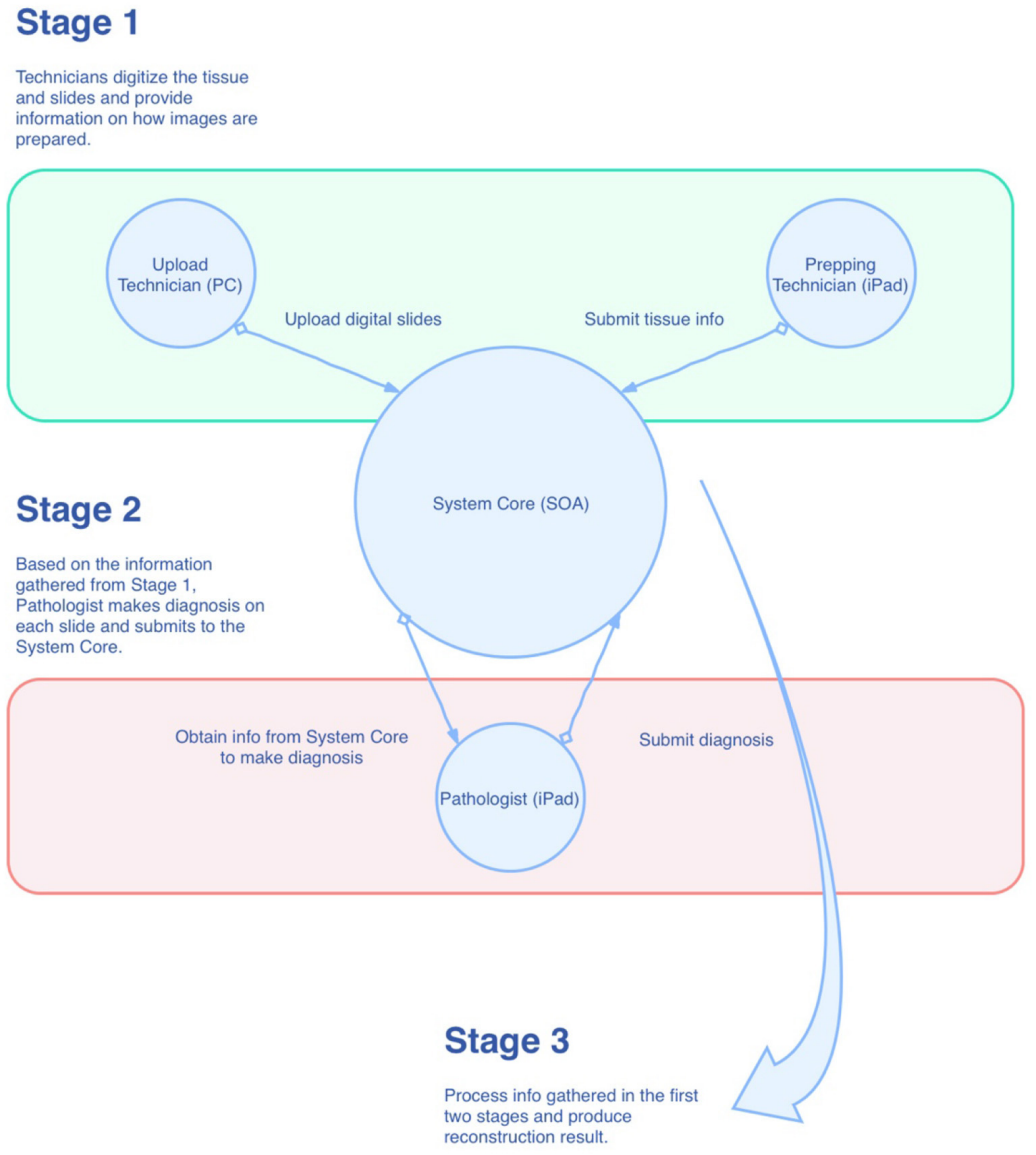
Overview of the mapping system.

As shown in Figure 1, the mapping system was designed in the style of service-oriented architecture (SOA), where the main functions of the system are wrapped up as centralized services in the private cloud for other components of the system. Conceptually, there are three roles within the system that have to work together for the reconstruction task. They are “Prepping Technician,” “Upload Technician,” and “Pathologist,” respectively. As just mentioned, these roles are conceptual, which means they can be played by one single individual or can be played by multiple individuals. During the process, information is collected at the System Core and then passed to the role that requires it. Eventually, all the information is gathered and processed in the System Core to produce the final result. The reconstruction algorithm can be broken down into the following steps: (1) foreground detection, (2) segregation of strips, and (3) linear mapping. We will describe these steps in more detail in the following sections.

### Foreground Detection

We adopted Otsu's method, a widely accepted foreground detection method in computer vision, to perform this task. To be more specific, a grid search of the thresholding parameter t was done on the grayscale digital slide thumbnail (which will be called the slide from now on) to reduce the intra-class variance.

With the searched threshold t^*^, we could turn the grayscale image into a binary image where the 1s mark the locations of the foreground pixels.

### Segregation of Strips

When a slide is prepared properly, the tissue strips will lie parallel to one another. Based on this assumption, the segregation algorithm can be broken down into the following steps: (1) auto tilting; (2) denoising: erosion and dilation; (3) denoising: Suzuki and Abe topological analysis; and (4) segregation of centroids.

We assumed the tissue strips were homogeneous within their boundaries. With this assumption, we could grid search within a range of angles to calculate the following for each angle:

1. Sum up projections onto the *y*-axis of the pixels;
2. Remove values <90th percentile;
3. Calculate the mean of the resulting values.

The target angle a^*^ is then obtained by computing the argmax for all the mean values. This algorithm helps to rotate the slide in a way so that all the tissue strips are horizontal.

Erosion and dilation are two fundamental operations in morphological image processing. The erosion operation uses a structuring element *B* (typically an all-one matrix) to move inside the target image *A* in a way that the center of B would cover every single pixel of *A* in turn. The transformed image *A'* satisfies *A* ⊖ *B* = ⋂_*b*∈*B*_*A*_−*b*_. In this research, we chose $B\;=\left(\begin{array}{c} 1\;1\;1\\ 1\;1\;1\\ 1\;1\;1 \end{array}\right)$.

The intuition of this is that when *B* is sliding across *A*, the submatrix of *A* that coincides with *B* has to include *B* (in the sense of set theory) so that the resulting value is 1, otherwise it would be zero. This operation would tend to zero out *scattered* values in the original image *A* since the neighborhood of those scattered values cannot form a submatrix that includes *B*. This also tells us that the larger the size of *B* we choose, the heavier the erosion operation.

Similarly, the dilation operation satisfies the following condition:


(1) $$\begin{array}{l} A⊕B=\cup_{b\in B}A_{b}, \end{array}$$

and interpolates extra values in between the scattered values. We use *B* = (1, 1, 1, 1, 1), and the dilation is applied twice in practice.

Then we used Suzuki and Abe's topological analysis to find the connected components with the maximum areas in order to denoise the image further. Since the number of strips on each slide is given (which is equal to the number of the cutting lines to be restored), let the number be *k*. We can retain *k* connected components with the maximum areas, to avoid disturbance from any unanticipated noise. Furthermore, we can identify the centroids of these maximum areas for further computation.

Now that the slide is binarized, noise-free, and rotated so that the strips are horizontally aligned. We can find the midpoints between the centroids obtained from Suzuki and Abe's topological analysis, and hence the segregation of strips is done.

### Linear Mapping

In the gross picture, each cutting line can be described and identified with its endpoints. Each cutting line is a 1D the entity in a 2D linear space. For each cutting line, there is a corresponding stripe. Therefore, the relative coordinates of the lesion pixels (with respect to the pixels of the entire strip) can be projected to the *x*-axis, to obtain a 1D representation, removing their depth information. By normalizing it, we obtain a dimensionless parameter *t* for each lesion pixel. Let the endpoints of any cutting line be *p*_1_, *p*_2_, and their coordinates are (*x*_1_, *y*_1_) and (*x*_2_, *y*_2_), respectively. Then, for any normalized lesion pixel parameter *t*, we can calculate their corresponding coordinates in the gross picture linear space by


(2) $$\begin{array}{l} \{\begin{array}{l} x=\left(x_{2}-x_{1}\right)\;t+x_{1}\\ y=\left(y_{2}-y_{1}\right)\;t+y_{1\cdot } \end{array} \end{array}$$

### System and Process Design

In the mapping system, we break down the entire reconstruction process into smaller tasks and assign these tasks to three different roles: Prepping Technician, Upload Technician, and Pathologist. We make use of the iPad to accelerate the tissue preparation and slide diagnostic process. Similar methodologies can also be applied to other platforms. The tasks are enumerated by each role in the following:

- Prepping Technician (on iPad):
  1. Capture an image of the prepared tissue;
  2. Trace the cutting lines with the Apple Pencil to record the line coordinates of the lines in the tissue space;
  3. Arrange the lines into groups; the lines represent the strips, and the groups represent the slides on which the strips will be placed;
  4. Save and submit.
- Upload Technician (on PC):
  1. Scan the slides;
  2. Select the digital images of the slides;
  3. Save and submit.
- Pathologist (on iPad):
  1. Make diagnoses and uses the Apple Pencil to make annotations on the slides;
  2. Perform corrections (flipping, rotation, auxiliary lines) to the slides;
  3. Submits and completes the reconstruction.

Once the pathologist submits the diagnosis result, the System Core will integrate all the data collected from the three roles and calculate the final result. The result will then be available for viewing by the pathologist.

## Results

The size of the specimens ranged from 1.7 × 1.4 to 8.4 × 5.2 cm. At our center, we used to put 3–5 tissue strips in each block. The smallest specimen had two blocks and seven tissue strips, while the largest specimen had 34 blocks and 81 tissue strips. The time required for mapping depends on the size of the specimen and the number of strips that contain tumors (i.e., tumorous strips). In addition, the complexity of the tumor area outline also affected the consumed time. Among the 23 specimens, the number of tumorous strips in a single specimen was 1–36, and the median was 12.

**Figure 2:**
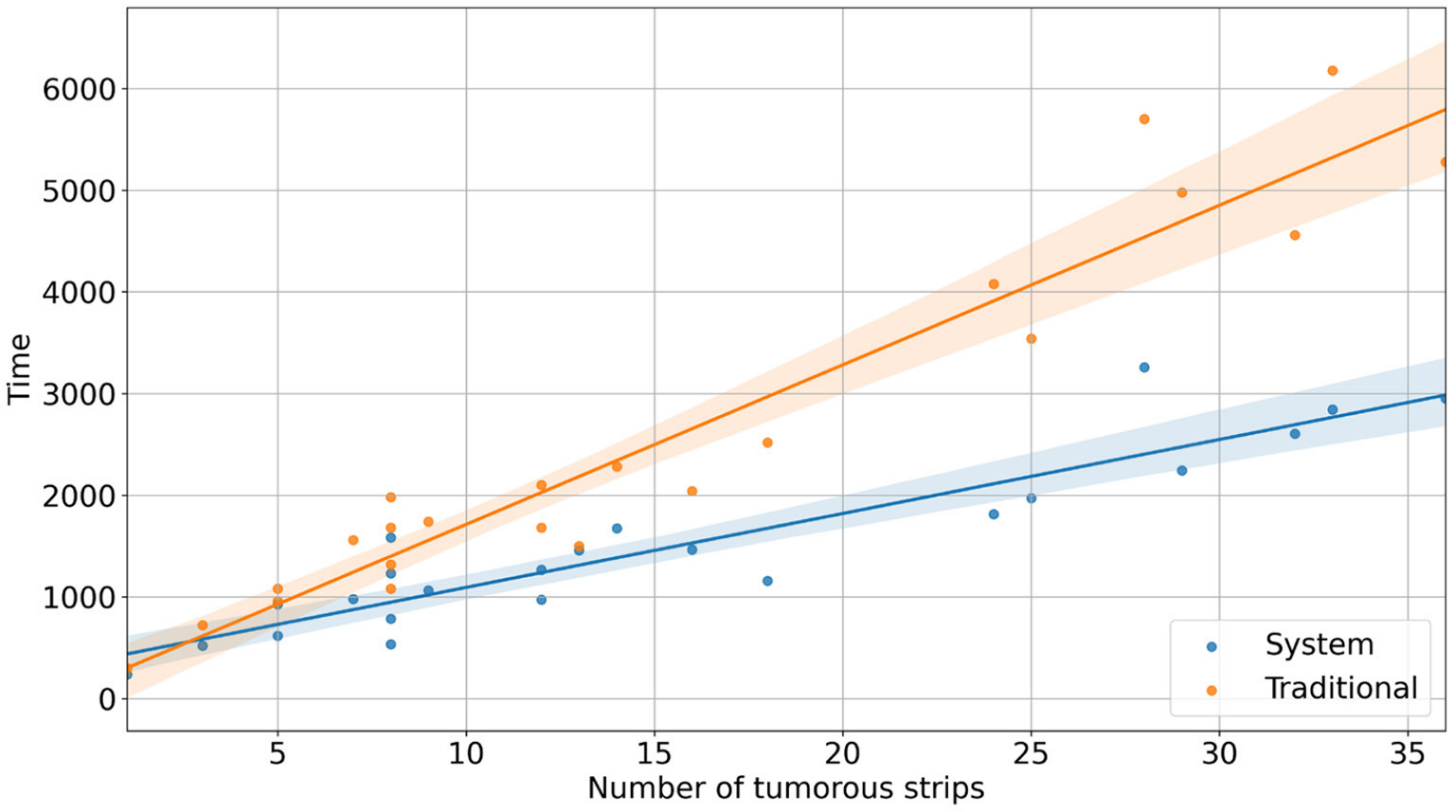
The diagnostic time for cases with different numbers of tumorous strips.

**Figure 3:**
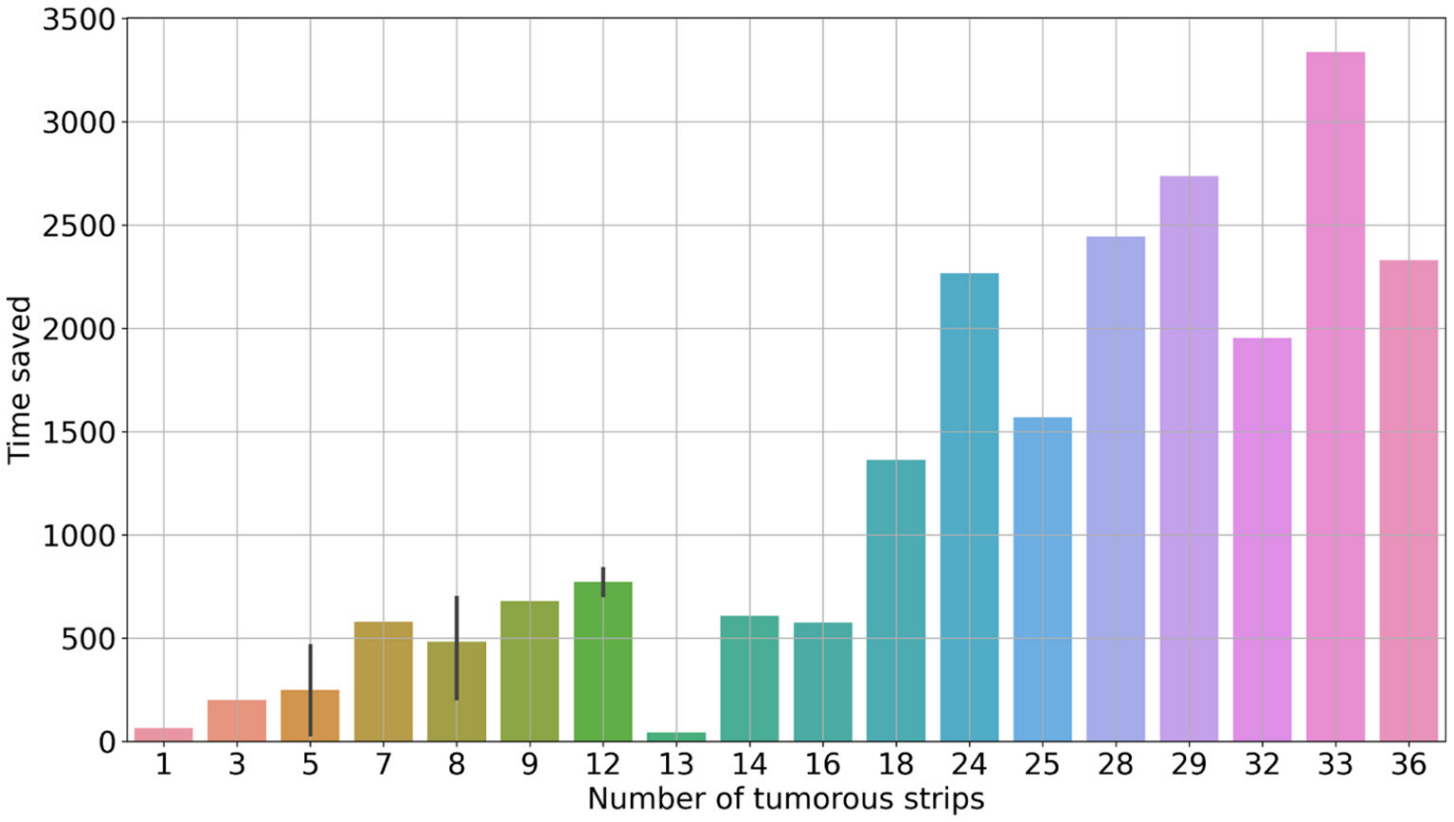
Saved time for diagnosing the entire case against the number of tumorous strips.

**Figure 4:**
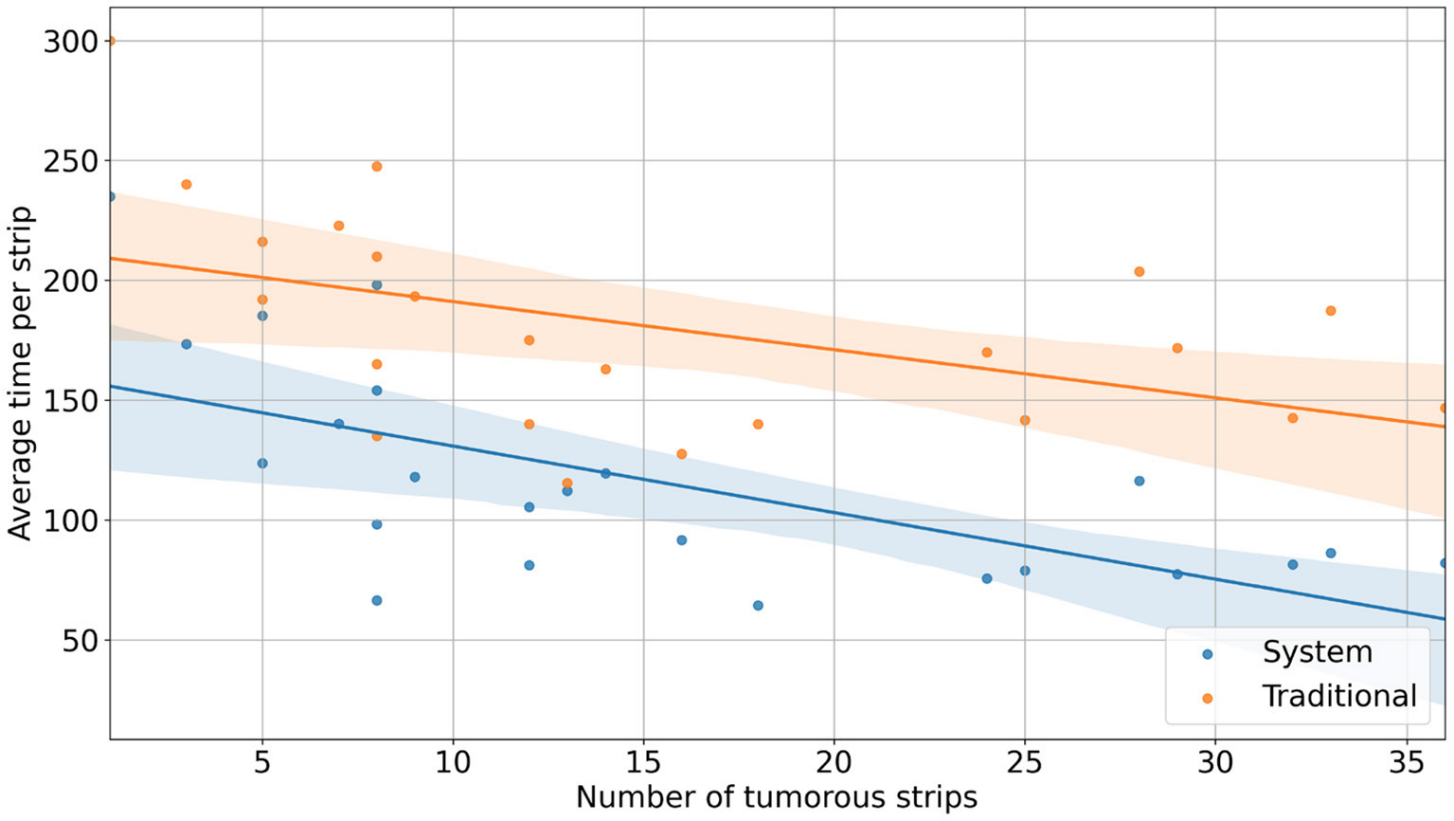
Average strip diagnostic time vs. the number of tumorous strips.

With the reconstruction system, the diagnostic time for a single case ranged from 235 s (3 min 55 s) to 3,257 s (54 min 17 s), with a median time of 1,265 s (21 min 5 s). In comparison, with traditional methods, the mapping time for a case ranged from 300 s (5 min) to 6,180 s (103 min), with a median time of 1,980 s (33 min) (Figure 2). Using the system, the time saved for a single case ranged from 34 s to 3,336 s (55 min 36 s) (Figure 3). Although specimens of larger and more complex tumor areas took more overall time, the average diagnostic time for tumorous strips was relatively less. The average diagnostic time of a tumorous strip was 64.3–235 s and 115.4–300 s, respectively, by systematic and traditional methods (Figure 4).

**Figure 5:**
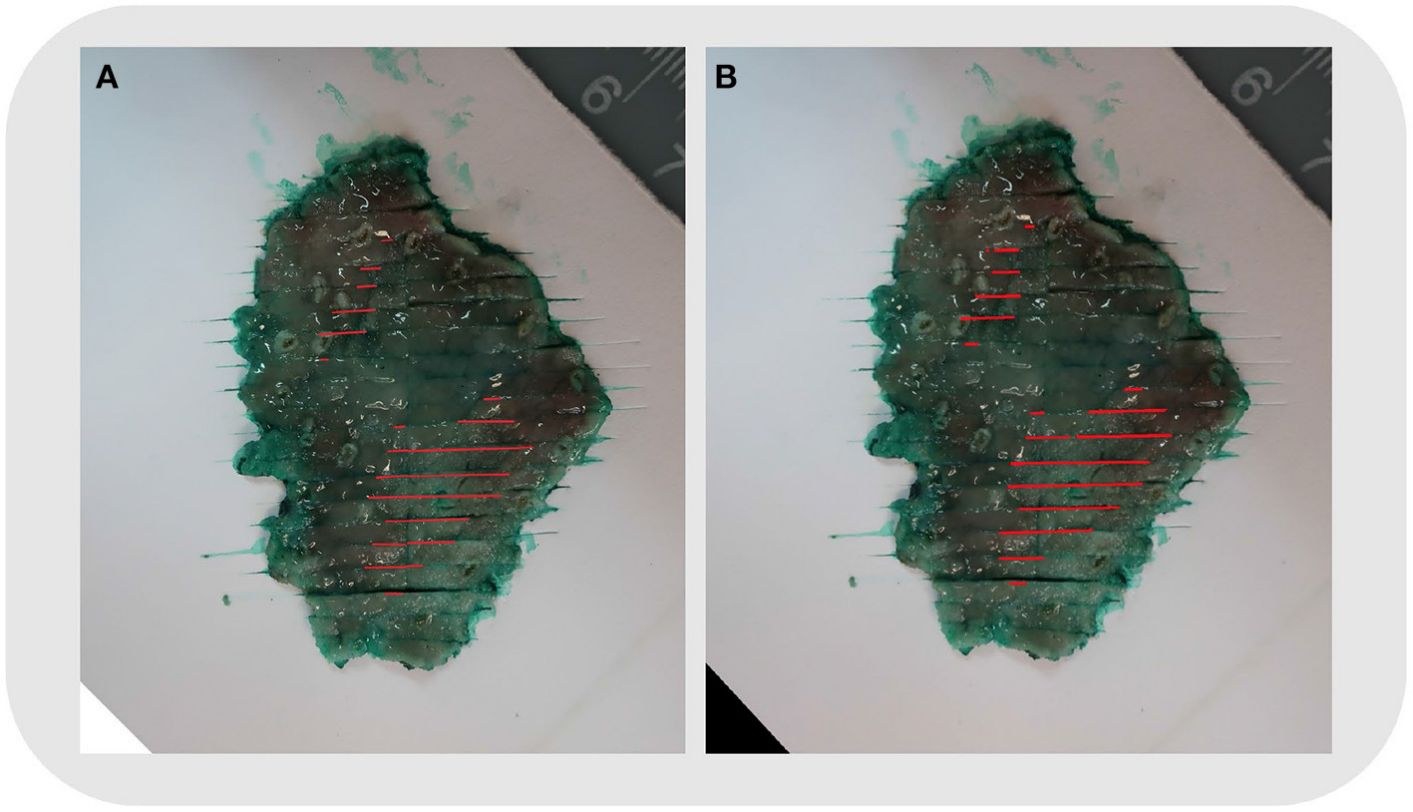
Case No. 5: **(A)** Mapping using the traditional method. **(B)** Mapping by the system. The shape, size, and location of the tumor area were the same. The reconstructed map showed two foci of the tumor clearly.

**Figure 6:**
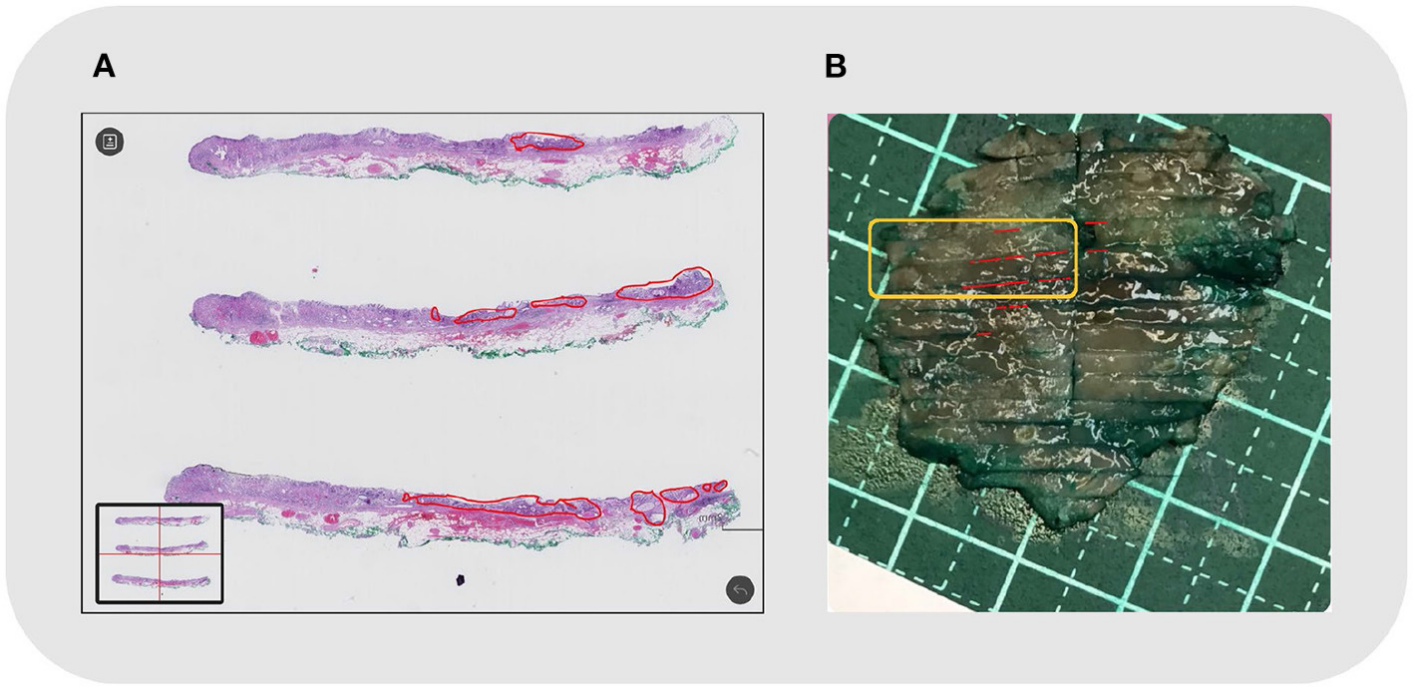
Case No. 20: **(A)** Local discontinuous lesions under microscopy, outlined in red; **(B)** The reconstructed map. The corresponding tissue strips in the orange frame showed discontinuous lesions in small foci.

The shape, size, and location of the tumor area were all controlled while comparing the two methods (refer to Figure 5). The pathologist marked the area of the lesion on the digital slides so that different tissues and lesions can be reconstructed. Traditionally, when looking at the tissues one by one, it was difficult to determine whether the discontinuous lesions were caused by irregular margins or by multifocal lesions. With the system, irregular or multifocal lesions were clearly shown (Figure 5). In addition, the system was able to accurately show small or discontinuous lesions that were hard to draw with traditional methods (refer to Figure 6).

## Discussion

Endoscopic submucosal dissection was first developed in Japan in the late 1990s. It has now been widely used to remove GI mucosal neoplasms to avoid local residue and recurrence (13). In addition, it has been indicated to minimize the chance of lymph node metastasis. The size of the lesion indicates the risk of lymph node metastasis (14). Oversized cancers or high-risk lymph node metastases require further surgery to ensure complete removal of the tumor. Maintenance of proper orientation, macroscopic description, and mapping are necessary to accurately determine the size of the lesion (4). Mapping and rebuilding the lesion on the gross photograph with cutting lines is recommended to evaluate the size and shape of the lesion, as well as to compare microscopic, macroscopic, and endoscopic findings (7, 15).

Traditionally, we could only reconstruct the lesion manually. We need to mark the lesion area on the slides and draw it in proportion to the cutting lines of the gross photograph. Tissue shrinks during the process of preparation, and the photos may have different magnifications to adapt to the size of the specimen. As a result, the corresponding marking process is tedious and time-consuming. The limitation of time constraints for endoscopists and pathologists may be the reason for hindering the implementation of this concept in daily routine practice (7).

Both endoscopic and pathological diagnosis are based on the observation of the morphology of lesions, which are closely related to each other and require good cooperation. In recent years, AI has made rapid progress and achieved amazing results in both fields. Now, AI is used in endoscopic diagnosis to identify early esophageal and gastric cancers (8, 16), colorectal polyps (9), and inflammatory bowel disease classification (11). AI techniques have also been developed and are widely utilized in research and the practice of surgical pathology (17). Across GI and liver cancer types, AI can automatically detect tumor tissues, capture prognostically relevant tissue features, and predict molecular and genetic alterations (11).

Based on our previous study (12), we established our AI-assisted topographic mapping system to make the mapping process automatic. For the 23 cases we studied, the system reconstructed them accurately. When compared with the traditional method, the shape and size of the lesions are the same. Without mapping, the size of the lesion could only be estimated from the tumor on the cutting line and the thickness of the accumulated tissue strips. If the cutting line is not parallel to or perpendicular to the long axis of the lesion, the true maximum diameter of the lesion cannot be obtained. The exact diameter can only be measured once the mapping is done accurately.

The system shows significant work efficiency improvements. The reconstruction time varies depending on specimen size and tumor area. Among our 23 cases, the system saved 34 s to around 55 min compared to the traditional method. Most specimens could be reconstructed within 20–30 min using the system. As traditional methods take a long time to get accurate results, only specific cases requiring discussion are studied. It is difficult to reconstruct each case. With the system, the reconstruction time is significantly reduced, making it applicable in daily work.

The system is well-demonstrated for multifocal and irregular lesions. Not all lesions are circular or oval, and some have very uneven boundaries. In such lesions, a cutting line may separate them into intermittent lesions. If not reconstructed, they may be considered multiple lesions. However, once the reconstruction is completed, it may be possible to find the connected regions to determine whether it is a single irregular lesion or a multifocal lesion. Among the three lesions (case nos. 13, 17, and 19) having irregular boundaries, we find local boundaries exceeding the contour by at least 0.5 cm. In addition, there were three cases (case nos. 6, 11, and 14) with two lesions. The topographic mappings from the system reveal them accurately.

The system can be more precise than the traditional method for small discontinuous areas. When the lesion area is very small, it is difficult to annotate on the glass slide, and could not be reconstructed by traditional methods. The digital slide can be enlarged and annotated, circling the small lesion area clearly. Meanwhile, at the edge of the lesion, the local morphology is very important to be compared with the endoscopic manifestations. A good topographic map can improve the understanding of the morphology of the lesion, especially for endoscopists (7). Based on a precisely performed reconstruction map, when comparing histology with endoscopic details, endoscopists would get feedback on the morphology of the lesions, promoting the understanding of the morphology under endoscopy and improving the diagnostic ability (7). After the implementation of the system, we carry out the topographic map for each case in our daily work. Moreover, it is applied in our monthly endoscope-pathology discussion.

The system is designed to be user-friendly and suitable for different tissue preparation routines. When the specimen is too large to fit into the embedding box, the tissue strips are disconnected, resulting in the splicing of cutting lines. Besides, the number of tissues inside each embedding box varies. Under all these situations, the system could be implemented directly without special settings or modifications. All that the system needs are clear gross pictures, digital slides, and the correct sequence of tissue strips.

The system has some defects. First, our limited number of cases at present only indicates the availability of the system. More practice may show specific problems that need to be improved. Second, for the automatic recognition of cutting lines, it is sometimes not so accurate under different photo color configurations. When the gross picture reflects strongly or the color is dark, the algorithm cannot recognize the cutting lines accurately. We are going to standardize the process of taking gross pictures and improve the model accuracy with more training cases. Moreover, we plan to further develop measures such as depth of infiltration, which is also an important parameter to indicate further surgery. In addition, our system can be extended to other tumor measurements. The tumor-node-metastasis (TNM) staging of particular tumors is based on the maximum tumor diameter. Accurate reconstruction and measurement of tumor size could better indicate the prognosis. We will add these functionalities to the system in future work.

A precise topographic map could reconstruct a lesion accurately. It not only makes the pathological report more accurate but also tightly connects histology with endoscopy. Therefore, it should be one part of the standardized protocol of ESD specimen pathology (7). The AI-assisted topographic mapping system completes the reconstruction process automatically, reducing the diagnostic work from several hours to half an hour. It not only improves work efficiency but also enables detailed and quantitative diagnosis that ultimately benefits patients.

## Data Availability Statement

The original contributions presented in the study are included in the article/supplementary material, further inquiries can be directed to the corresponding authors.

## Ethics Statement

The studies involving human participants were reviewed and approved by the Ethics Committee of the Peking Union Medical College Hospital (CAMS). Written informed consent for participation was not required for this study in accordance with the national legislation and the institutional requirements.

## Author Contributions

WZ was responsible for the design and overall implementation of the study. YX, ZS, SZ, and YY are responsible for pathological interpretation and calibration of lesions. SW and CK are responsible for artificial intelligence algorithm implementation and system construction. JC, XX, and WH were responsible for sampling, general photography, and section making and scanning. XW provides assistance in endoscopic resection and comparison. All the authors contributed to the article and approved the submitted version.

## Funding

This study was supported by the CAMS Innovation Fund for Medical Sciences (CIFMS) (Grant Nos. 2020-I2M-C&T-B-038 and 2018-I2M-AI-008).

## Conflict of Interest

SW was the co-founder, chief technology officer, and equity holder of Thorough Images. CK was the algorithm researcher of Thorough Images. The remaining authors declare that the research was conducted in the absence of any commercial or financial relationships that could be construed as a potential conflict of interest.

## Publisher's Note

All claims expressed in this article are solely those of the authors and do not necessarily represent those of their affiliated organizations, or those of the publisher, the editors and the reviewers. Any product that may be evaluated in this article, or claim that may be made by its manufacturer, is not guaranteed or endorsed by the publisher.
